# Guided Wave Phase Velocity Dispersion Reconstruction Based on Enhanced Phased Spectrum Method

**DOI:** 10.3390/ma15041614

**Published:** 2022-02-21

**Authors:** Vykintas Samaitis, Liudas Mažeika

**Affiliations:** Prof. K. Baršauskas Ultrasound Research Institute, Kaunas University of Technology, K. Baršausko St. 59, LT-51423 Kaunas, Lithuania; liudas.mazeika@ktu.lt

**Keywords:** guided waves, mechanical properties, phase velocity, non-destructive testing, composites

## Abstract

Fibre-reinforced composite laminates are frequently used in various engineering structures, due to their increased weight-to-stiffness ratio, which allows to fulfil certain regulations of CO_2_ emissions. Limited inter-laminar strength makes composites prone to formation of various defects, which leads to progressive degradation of residual strength and fatigue life of the structure. Using ultrasonic guided waves is a common technique for assessing the structural integrity of composite laminates. Phase velocity is one of the fundamental characteristics of guided waves and can be used for defect detection, material property estimation, and evaluation of dispersion. In this paper, a phase velocity reconstruction approach, based on the phase-shift method, was proposed, which uses frequency sweep excitation to estimate velocity at specific frequency harmonics. In contrast to the conventional phase spectrum technique, the proposed approach is applicable to the narrowband piezoelectric transducers and suitable for the reconstruction of dispersion curves for direct, converted, and multiple co-existing modes with high accuracy. The proposed technique was validated with finite element simulations and experiments, both on isotropic and anisotropic structures, analysing the direct, converted, and overlapped modes. The results demonstrated that, using the proposed technique, the phase velocity dispersion can be reconstructed at −20 dB level bandwidth of the transducer, with a relative error of ±4%, compared to the theoretical velocity predictions.

## 1. Introduction

The composites market is one of the strategic development areas of the European Union, which aims to strengthen their competitiveness and extend the use of composites in the sectors of aerospace, automotive, and renewable energy [1]. The current EU demand of carbon fibre is estimated to be 35% of the global demand, and it will have an annual growth of 10–12% [1,2], while the UK market will grow from 2.5 billion up to 10 billion pounds a year by 2030 [3,4,5]. Fibre-reinforced composite laminates can offer increased strength- and stiffness-to-weight ratios, which allow for meeting the demanding requirements of CO_2_ emissions. However, composites have limited interlaminar strength and are prone to formation of fibre breakage, matrix cracking, delaminations, porosity, and other structural defects. Such defects are usually hidden and progressively degrade the residual strength and fatigue life, eventually leading to sudden structural failure. Using ultrasonic guided waves is a common method for periodic inspection and monitoring of structural integrity of plate-like composite laminates, that offers large inspection areas and sensitivity to structural damage of various kinds [6,7,8]. To date, many studies are available that employ guided waves for the detection and quantification of impact damage [9,10,11,12], delaminations [13,14,15,16,17], and other defects in composite laminates. Guided wave propagation in composites is determined by many factors, including, but not limited to, multi-layered structure and anisotropy, object boundaries, dispersion, multiple co-existing modes, and mode conversion. Phase velocity is one of the fundamental properties of guided wave modes that depends on composition, structural integrity, elastic properties, and frequency-thickness product of composite. Velocity measurements can be exploited both for material characterisation and damage detection, offering several benefits, such as validation of material properties, identification of wave-packets in complex guided wave signals, and sizing of defects [18,19,20].

However, reconstruction of phase velocity from overlapped, multimodal signals, and multi-layered anisotropic structures has been a long-standing problem. Initial phase velocity measurement approaches used threshold, zero crossing, or cross-correlation methods to evaluate the time-of-flight (ToF) of well-isolated guided wave modes [21]. The threshold method, in its simplest form, captures the time instance at which the signal crosses certain amplitude level. As these methods are based on signal amplitude, they are susceptible to noise and any other variation of the signal shape; hence, more advanced threshold-based ToF evaluation methods, such as variable ratios or similarity-based double threshold, were proposed [22,23]. The zero-crossing method seeks to obtain time instances at which the amplitude of the signal is equal to zero. To improve the accuracy of ToF estimation, using the zero-crossing technique, and avoid cycle skip problems, multiple zero-crossing points are being estimated within the same signal [24]. It is known that zero-crossing technique suffers from the phase uncertainty, especially at large propagation distances and under significant dispersion, as it become impossible to follow signal phase of the elongating wave packet and to avoid the cycle-skip. Recently, a technique based on zero-crossing and spectrum decomposition was proposed which exploits signals measured at sufficiently close distances and estimates the phase velocity, based on zero-crossing evaluation on signals filtered with different bandpass filters [25]. Cross-correlation technique is based on the measurement of correlation lag, between the received and reference signals. Such technique is considered suitable for low signal-to-noise ratio (SNR) signals, while the ToF accuracy mainly depends on the sampling ratio [26]. However, it is reported that cross-correlation-based ToF estimation may become significantly biased while analysing signals distorted due to scattering or dispersion [27].

The abovementioned ToF estimation methods can effectively be used for well-isolated and undistorted signals; however, they usually fail in analysing the overlapped, multimodal, scattered, and dispersed responses. Model-based approaches can partly deal with this problem by solving multi-dimensional and non-linear optimisation problems, while fitting synthetic signals to a segment of ultrasonic structural response. By using matching pursuit, chirplet transform, empirical mode decomposition or wavelet methods it is possible to decompose multimodal signals and to estimate their properties, such as frequency or ToF [28,29,30,31,32]. However, model-based methods are usually computationally expensive, as transformations are calculated in multi-dimensional space, while the selection of the mother wavelet or atoms is non-trivial task and may lead to unexpected results. It has been demonstrated that phase and group velocities can be reconstructed using phase-shift methods. First proposed by Sachse [33] and used by Schumacher [34], phase-shift methods are based on the estimation of the phase difference between transmitted and received signals, which is proportional to propagation distance. Initially, phase-shift methods were extensively used for bulk waves and later applied to laser-induced guided waves. In contrast to broadband laser-based excitation, piezoelectric sensors, that are more cost effective and commonly used in structural health monitoring applications, usually have quite narrow frequency band, due to the type of excitation, vibration mode, and size of the transducer; hence, the phase velocity reconstruction zone essentially becomes limited. Moreover, in order to avoid phase ambiguity, the distance between transmitted and recorded signals is required to be up to one wavelength, which limits spatial velocity distribution reconstruction capabilities.

In this paper, a phase velocity reconstruction approach is presented that uses phase-shift method and excitation frequency sweep to obtain phase velocity estimations in the entire band of transducer. Two sensors, positioned in close proximity, are used to record signals propagated through the structure and estimate the phase-shift between the signals. At each excitation frequency, the reconstruction of phase velocity is performed at specific frequency components only, which correspond to the peak values of the magnitude spectra. These peak frequencies depend on the frequency response of the excitation signal; hence, phase velocity values can be collected at different frequencies, allowing us to achieve a wideband reconstruction. The validity of the approach is demonstrated through simulations and experiments by reconstructing the phase velocities of S_0_ and converted A_0_ modes, as well as identifying guided wave modes in complex multimodal signals.

In contrast to the classic phased spectrum method, the proposed approach allows to reduce the relative error of the phase velocity reconstruction from ±11% to ±4% and increase significantly the reconstruction bandwidth from −6 dB to −20 dB of the ultrasonic probe. As a result, using only two signals, measured in close proximity, the proposed phased spectrum method can achieve the reconstruction accuracy and bandwidth, which, to date, could be achieved only with techniques that include scanning of the sensor over a sufficiently large area.

## 2. Description of Proposed Phase Velocity Estimation Method

**Figure 1:**
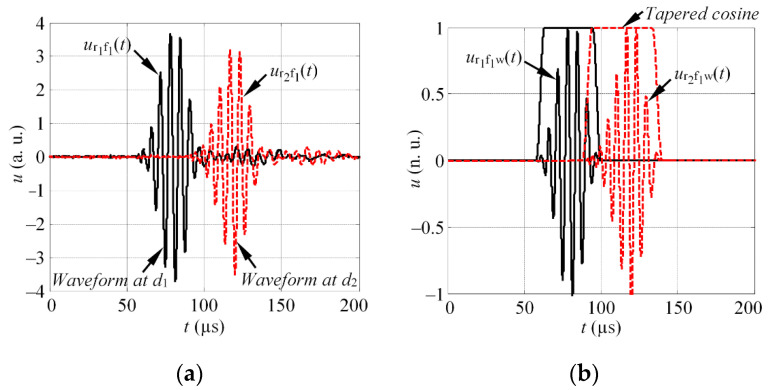
(**a**) The example of the waveform, registered with receivers *r*_1_ and *r*_2_, at distances *d*_1_ and *d*_2_; (**b**) the illustration of waveform windowing to isolate the wave packet of single mode.

The proposed phase velocity reconstruction approach employs a classic phase-shift method to estimate the velocity values at specific frequencies that correspond to peak values of the magnitude spectra of received signal. By repeating this procedure at different excitation frequencies, velocity values can be reconstructed at wide band, covering the entire bandwidth of the transducer. Variation of the excitation frequency allow different harmonics to be enhanced or suppressed, which is the key factor if reconstruction is performed at peak values of magnitude spectra only. The algorithm of the proposed method can be summarized with the following steps:

1. The transducer is driven by a burst at a central frequency of *f*_1_, and the waveforms *u*_r1f1_(*t*) and *u*_r2f1_(*t*) are registered with receivers *r*_1_ and *r*_2_, each positioned at a distances *d*_1_ and *d*_2_ from the source (see Figure 1a for reference).
2. The waveforms *u*_r1f1_(*t*) and *u*_r2f1_(*t*) are windowed using the tapered cosine window *w*(*t*) to isolate the wave packets of particular mode (see Figure 1b):

(1) $$u_{r_{1}f_{1}w}\left(t\right)=u_{r_{1}f_{1}}\left(t\right)\cdot w\left(t-t_{1}\right),\;\;u_{r_{2}f_{1}w}\left(t\right)=u_{r_{2}f_{1}}\left(t\right)\cdot w\left(t-t_{2}\right)$$

where *u*_r1f1w_(*t*) and *u*_r2f1w_(*t*) represent the windowed versions of the waveforms *u*_r1f1_(*t*) and *u*_r2f1_(*t*), respectively; *t*_1_ and *t*_2_ correspond to the time instances of the maximum amplitude of the wave packet.

**Figure 2:**
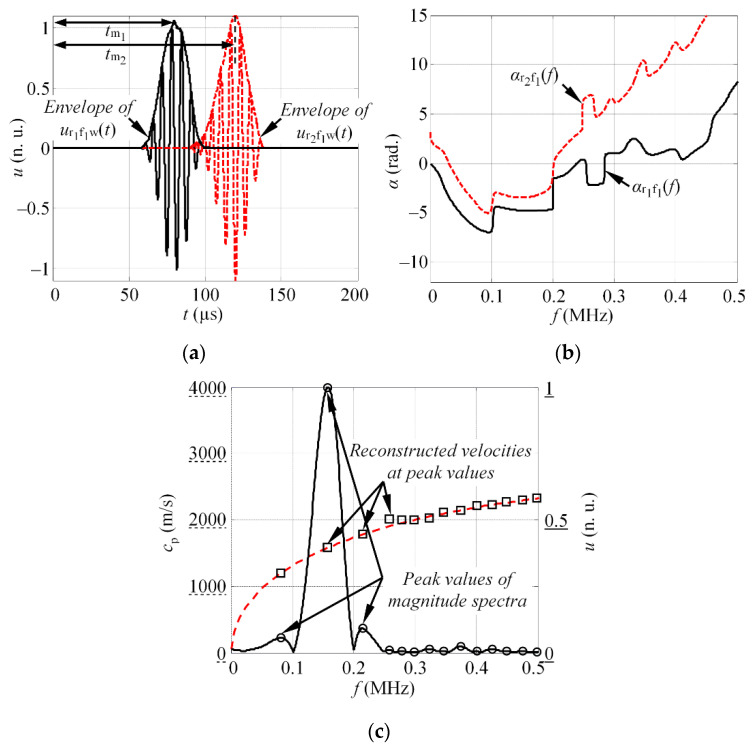
(**a**) The illustration of the shift of waveform in time domain to the maximum value of the Hilbert envelope; (**b**) the phase spectra of the waveforms, registered at distances *d*_1_ and *d*_2_; (**c**) the normalized magnitude spectra of the waveform, captured with receiver *r*_1_ with the local maximum frequency values (circle markers), at which the phase velocity values are estimated (square markers) (dashed line represents the theoretical DC).

3. Each waveform, *u*_r1f1w_(*t*) and *u*_r2f1w_(*t*), is shifted in the time domain by −*t*_m1_ and −*t*_m2_, to avoid the uncertainties in the phase unwrapping procedure. The waveforms can be shifted according to the centroid of signal [35] or maximum value of the Hilbert envelope [36], in case of moderate dispersion:

(2) $$u_{r_{1}f_{1}s}\left(t\right)=u_{r_{1}f_{1}w}\left(t+t_{m_{1}}\right),\;\;u_{r_{2}f_{1}s}\left(t\right)=u_{r_{2}f_{1}w}\left(t+t_{m_{2}}\right),$$

$$t_{m_{1}}=\arg\left(\underset{t}{\max}\left[\mathrm{HT}\left|u_{r_{1}f_{1}w}\left(t\right)\right|\right]\right),\;\;t_{m_{2}}=\arg\left(\underset{t}{\max}\left[\mathrm{HT}\left|u_{r_{2}f_{1}w}\left(t\right)\right|\right]\right),$$

where HT denotes the Hilbert transform; *t*_m1_ and *t*_m2_ are the time instances, which corresponds to the maximum of Hilbert envelope, in such a way that the influence of the signal delay due to phase velocity is compensated. The shift in time domain is illustrated in Figure 2a.

4. The complex frequency spectra of each time-shifted waveform, *u*_r1f1s_(*t*) and *u*_r2f1s_(*t*), is obtained employing the Fourier transform:

(3) $$U_{r_{1}f_{1}}\left(jf\right)=\mathrm{FT}\left[u_{r_{1}f_{1}s}\left(t\right)\right],\;U_{r_{2}f_{1}}\left(jf\right)=\mathrm{FT}\left[u_{r_{2}f_{1}s}\left(t\right)\right]$$

where FT represents the Fourier transform.

5. The phase difference Δ*ϕ*(*f*) between shifted signals *u*_r1f1s_(*t*) and *u*_r2f1s_(*t*), is estimated for a given frequency band *f* (see Figure 2b):

(4) $$\Delta \varphi_{f_{1}}\left(f\right)=\left(\alpha_{r_{1}f_{1}}\left(f\right)-\alpha_{r_{2}f_{1}}\left(f\right)\right),$$

αr1f1(f)=arctan[Im[Ur1f1(jf)]Re[Ur1f1(jf)]], αr2f1(f)=arctan[Im[Ur2f1(jf)]Re[Ur2f1(jf)]],

where Im and Re represent the imaginary and real of the complex Fourier spectra.

Note that the phases *α*_r1f1_(*f*) and *α*_r2f1_(*f*) are calculated in a range of [−π…π] radians. If the true phase of the particular frequency is less than −π radians, it will be represented below the π radians. This means that some discontinuities will appear, in case the phase goes beyond the ±π radian limit. Therefore, the phases *α*_r1f1_(*f*) and *α*_r2f1_(*f*) have to be unwrapped.

6. The phase velocity, as a function of frequency, is calculated at particular frequencies, *f*_1,*k*1_, using a modified version of the phase spectrum method:

(5) $$c_{p}\left(f_{1,k_{1}}\right)=\frac{2\pi f_{1,k_{1}}d}{\Delta \varphi_{f_{1}}\left(f_{1,k_{1}}\right)-2\pi f_{1,k_{1}}\left(t_{m_{1}}-t_{m_{2}}\right)},$$

where *f*_1,*k*1_ are the frequencies that corresponds to the peak values of the magnitude spectra |*U*_r1f1_(*jf*)| at excitation frequency *f*_1_; *k* = 1 ÷ *K*_1_, *K*_1_—is the total number of detected peaks at excitation frequency *f*_1_, and *d* is the separation distance between the receivers *r*_1_ and *r*_2_ (*d* = *d*_2_ − *d*_1_). The frequency selection for phase velocity estimation is illustrated in Figure 2c.

7. The intermediate values of the phase velocities at other frequencies are obtained by changing the excitation frequency to *f*_2_ and repeating the whole routine described above. The final result is obtained by combining the calculations at different excitation frequencies *f*_1_… *f*_N_:

(6) $$c_{p}\left(f\right)=\underset{f}{\mathrm{sort}}\left\{c_{p}\left(f_{1,k_{1}}\right),\;\ldots ,\;c_{p}\left(f_{n,k_{n}}\right),\ldots ,c_{p}\left(f_{N,k_{N}}\right)\right\},$$

where *N* is the number of excitation frequencies used to drive the emitter.

The method presented above is applicable to flat structures with uniform thickness, which can be multi-layered, anisotropic, or isotropic. In contrast to the conventional phase spectrum method, it provides better accuracy of velocity estimation, which will be demonstrated in the subsequent Chapter.

## 3. Experimental Validation on Isotropic Samples

In this section, the proposed phase velocity estimation approach is validated with the appropriate experiments. For this purpose, the phase velocity values, extracted with the proposed approach, are compared with the theoretical calculations, which were considered a reference. In this study, the velocities of the S_0_ mode in the aluminium sample will be analysed.

**Figure 3:**
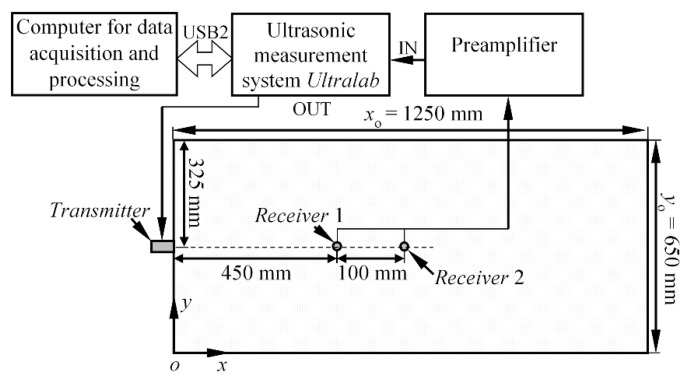
The schematic diagram of the experimental set-up for the validation of phase velocity estimation method.

The experiments were carried out on the aluminium alloy 2024 T6 plate, which was 2 mm thick, 650 mm wide, and 1250 mm long. The well-known isotropic material was deliberately selected for this study, in order to be able to compare the experimental results with the theoretically estimated values. The S_0_ mode was launched into the structure by attaching the thickness mode transducer to the edge of the Al plate, as is shown in Figure 3. For the reception, two transducers, *r*_1_ and *r*_2_, possessing the same characteristics, were bonded perpendicularly to the upper surface of the specimen at distances *d*_1_ = 450 mm and *d*_2_ = 550 mm from the source (see Figure 3).

**Figure 4:**
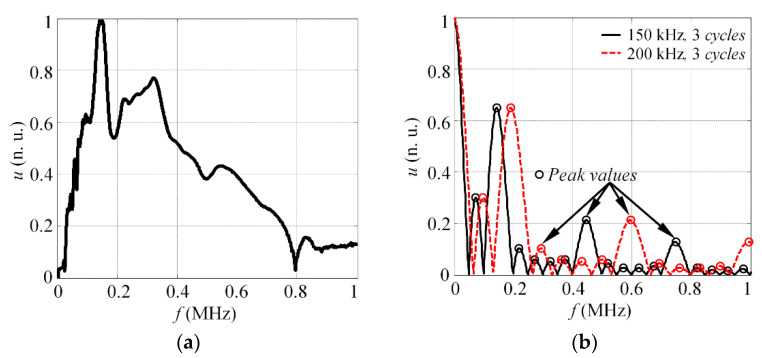
(**a**) The frequency response of the thickness mode transducer used for the experiments; (**b**) the magnitude spectra of three cycles (150 kHz) (solid line) and three cycles (200 kHz) (dashed line) square excitation pulse.

In this paper, transducers with a central frequency of 240 kHz and bandwidth of 340 kHz at −6 dB level were used. The frequency response of the probe can be seen in Figure 4a. To reconstruct the dispersion curve under the wide band, two different scenarios employing the square pulse excitation were used, as follows: *n*_1_ = 3 cycles, *f*_1_ = 150 kHz; and *n*_2_ = 3 cycles, *f*_2_ = 200 kHz. Such excitation frequencies were deliberately selected, according to the magnitude spectrum of excitation pulse, which can be seen in Figure 4b. The results, presented in the figure, demonstrate that a minor shift of excitation frequency from 150 to 200 kHz enables peak amplitudes of the magnitude spectra to be obtained at different frequencies. Moreover, the local maximum values, in case of 200 kHz excitation, mostly correspond to the local minimum frequencies of 150 kHz excitation. Thus, excitation under the selected frequencies enables a large variety of reconstruction frequencies to be obtained. In this case, it was presumed that the selected excitation frequencies will provide a sufficient amount of velocity values. In other cases, more excitation frequencies may be used, exploiting the whole bandwidth of the transducer.

**Figure 5:**
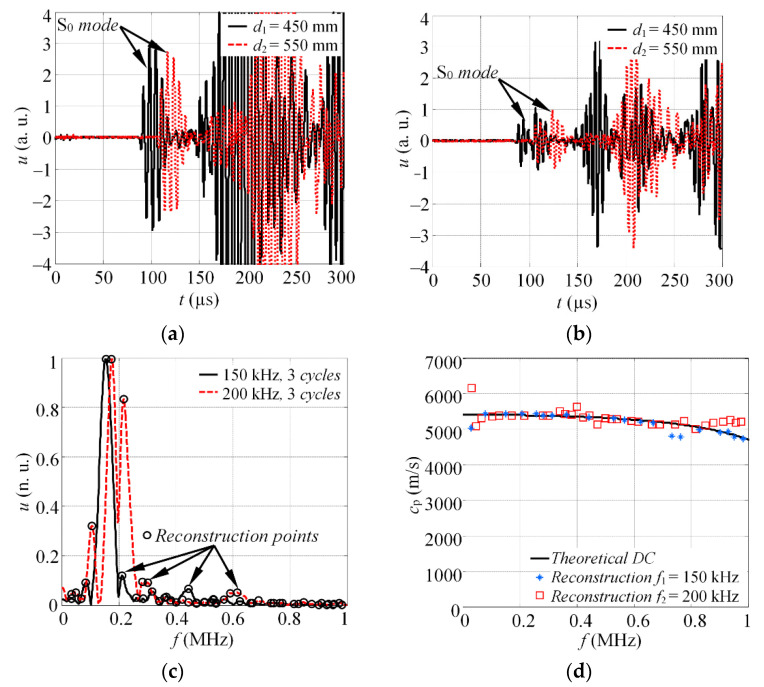
The experimental waveforms of the S_0_ mode, at distances *d*_1_ and *d*_2_, in case of (**a**) 150 (**b**) and 200 kHz excitation; (**c**) the magnitude spectra of windowed S_0_ mode at different excitation frequencies; (**d**) the combined reconstruction of dispersion relations.

The experimental waveforms of the S_0_ mode, at distances *d*_1_ and *d*_2_, under the *f*_1_ = 150 and *f*_2_ = 200 kHz excitation, are presented in Figure 5a,b, respectively. The magnitude spectra, |*U*_r1f1_(*jf*)| and |*U*_r2f2_(*jf*)|, of the windowed S_0_ mode wave packet can be seen in Figure 5c. The frequencies at which the phase velocity values were extracted are indicated with circle markers. Finally, the reconstructed dispersion curve of the phase velocity for the S_0_ mode, along with theoretical estimation, is shown on Figure 5d. The theoretical dispersion curve was calculated by employing the SAFE method and material properties of aluminium 2024 T6 (the density: *ρ* = 2780 kg/m^3^; Young’s modulus: *E* = 72 GPa; Poisson’s ratio: *υ* = 0.35).

The results in Figure 5d show that the phase velocities are reconstructed in the frequency band up to 0.8 MHz. According to the frequency response of the transducer used in this study (see Figure 4a), the technique enables the phase velocities in the −20 dB level bandwidth of the actuator to be reconstructed. In this study, a total of *K* = 52 velocity values were extracted at a band up to 1 MHz. This means that using two frequencies to drive the transducer, 52 reconstruction points were observed that correspond to peak frequencies of the magnitude spectra. Such a number of reconstruction points is relative and depends on the total number of excitation frequencies, *N*, and obtained number of peak values of magnitude spectra, in case of each excitation frequency.

It is noteworthy that the general reliability of the phase spectrum method depends on the proper selection of the time window to crop the wave packet of the single mode for FFT. The proposed method implicitly assumes that only one mode is present at the selected time window.

**Figure 6:**
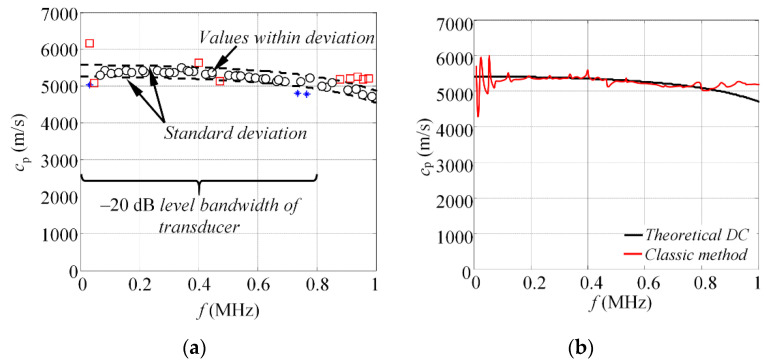
(**a**) The graphic representation of standard deviation, showing the spread of estimated phase velocity values and (**b**) reconstruction of phase velocity dispersion curves using the classic phased spectrum method.

In order to estimate the agreement of the results with theoretical phase velocities, the standard deviation (STD) was used as a measure of spread:

(7) $$\sigma =\sqrt{\frac{1}{K_{1}-1}\overset{K_{1}}{\underset{i=1}{\sum }}{\left|\left(c_{p}\left(f_{i}\right)-c_{t}\left(f_{i}\right)\right)-\mu \right|}^{2}},$$

(8) $$\mu =\frac{1}{K_{1}}\overset{K_{1}}{\underset{i=1}{\sum }}\left(c_{p}\left(f_{i}\right)-c_{t}\left(f_{i}\right)\right),\;$$

where *K*_1_ is number of points in reconstructed phase velocities, *c*_p_(*f_i_*) is a vector of reconstructed phase velocity values, and *c*_t_(*f_i_*) are the corresponding reference phase velocity values, calculated using the SAFE method. The estimated standard deviation of the calculated phase velocity values is *σ* = 161 m/s. This leads to the conclusion that 40 out of 52 velocity values (77%) are within the standard deviation range, as shown in Figure 6a.

The experimental results, presented in this section, demonstrate that proposed approach reconstructs the phase velocity values at frequencies up to 800 kHz for the selected probe. At frequencies above 800 kHz, the approach starts to fail at capturing the pattern of the dispersion curve. Hence, it can be said that the phase velocity values of the S_0_ mode can be reconstructed at −20 dB bandwidth or 0.1 level of the transducer, according to its normalized magnitude spectra, presented at Figure 4a. The standard deviation of the reconstructed phase velocities, calculated according to Equation (7), is 161 m/s, which provides relative error of phase velocity estimation equal to ±3% for the S_0_ mode, calculated according to:

(9) $$\delta =\left(\frac{\sigma \times 100\%}{\mu_{c_{t}\left(f\right)}}\right),$$

where *µ_c_*_t(*f*)_ is the mean theoretical phase velocity value in the selected frequency band under analysis.

In order to emphasize the achieved improvement, the signals of the S_0_ mode, obtained at 200 kHz, were processed using classic phased spectrum method, described in [33,34]. The reconstructed phase velocity curve is presented at Figure 6b. The results indicate that highest velocity reconstruction accuracy can be obtained at frequency band 200–340 kHz, which corresponds to −6 dB bandwidth of the sensor. The standard deviation of the S_0_ mode phase velocity reconstruction is estimated to be 592 m/s for the classic phase spectrum method, which gives ±11% relative phase velocity reconstruction error. In can be concluded that proposed approach allow to increase the reconstruction bandwidth, from 140 to 800 kHz, and reduce the relative velocity estimation error, from ±11% to ±3%, for the S_0_ mode.

## 4. Identification of Converted Modes

In this section, the numerical validation of the proposed phase velocity reconstruction method will be presented. The major focus will be given to the method performance, in case the analysed signal is surrounded by the wave packets of other co-existing modes. To achieve the purpose of this study, the phase velocities of the converted A_0_ mode will be analysed, which convert from the S_0_ mode, due to the presence of notch.

**Figure 7:**
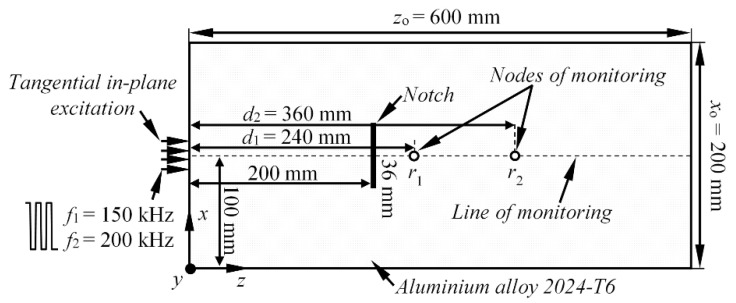
Illustration of the set-up of numerical aluminium plate FE model with the notch.

To fulfil the scope of this research, the 3D linear structural mechanics finite element model of isotropic aluminium alloy 2024 T6 plate (600 × 200 × 2 mm) is considered. The top view of the analysed structure is presented on Figure 7. The S_0_ mode was initially launched into the structure by applying the in-plane force to the shortest edge of the Al plate. To generate the converted A_0_ mode, the vertical 36 mm wide (along *x* axis) crack-type defect, with a depth of 66% of the plate thickness, was introduced by duplicating the nodes of the mesh. In such way, a complete disbond was simulated, without changing the shape of finite element model. It was shown by the various researchers that, if a crack is not symmetrical to the middle plane of the plate, according to the thickness, the mode conversion takes place upon the wave interaction with the notch, and both the S_0_ and A_0_ modes are expected as the reflected and transmitted waves [37]. The defect was centred, with respect to the short edge of the sample, and situated at the distance of 200 mm from source of Lamb waves (see Figure 7).

**Figure 8:**
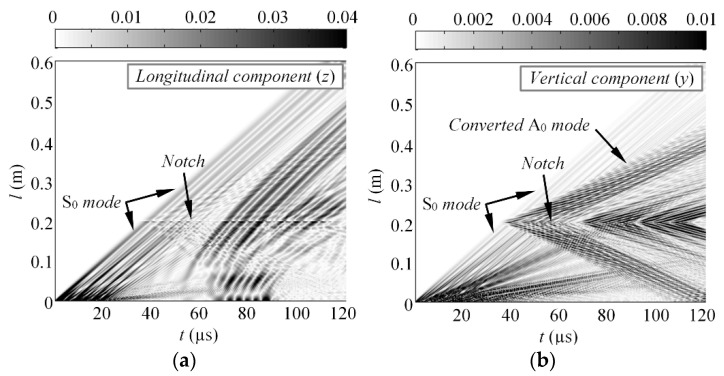
The (**a**) longitudinal and (**b**) vertical component of particle velocity along the centreline of the sample, in case of 150 kHz excitation.

Throughout the simulations, the ANSYS 17.1 implicit solver and 3D structural solid solid64 finite elements were used, which are defined by eight nodes having three degrees of freedom at each node and 2 × 2 × 2 integration points. The finite elements were hexahedrons, meshed using structured grid. Once again, two different scenarios employing the square pulse excitation were used, as it was described in the previous section. At first, the excitation pulse consisted of *n*_1_ = 3 cycles and a central frequency of *f*_1_ = 150 kHz. Meanwhile, in the second case, the Lamb waves were excited with *n*_2_ = 3 cycles at *f*_2_ = 200 kHz. The average mesh size was equal to 0.5 mm, which corresponds to 21 nodes per wavelength for the slowest A_0_ mode at *f*_1_ and 17 nodes per wavelength at *f*_2_. The integration steps in the time domain were 0.33 and 0.25 µs, respectively, which produces a 1/20 of the period, both at *f*_1_ and at *f*_2_. The variable monitored in this study was a vertical component of particle velocity (*y*) along the centreline of the sample. The waveforms for the phase velocity estimation were selected along the centreline of the sample at distances *d*_1_ = 240 mm and *d*_2_ = 360 mm. The B-scan images of the longitudinal (*z*) and vertical component (*y*) of the particle velocity, showing the S_0_ and converted A_0_ modes, are presented in Figure 8a,b.

**Figure 9:**
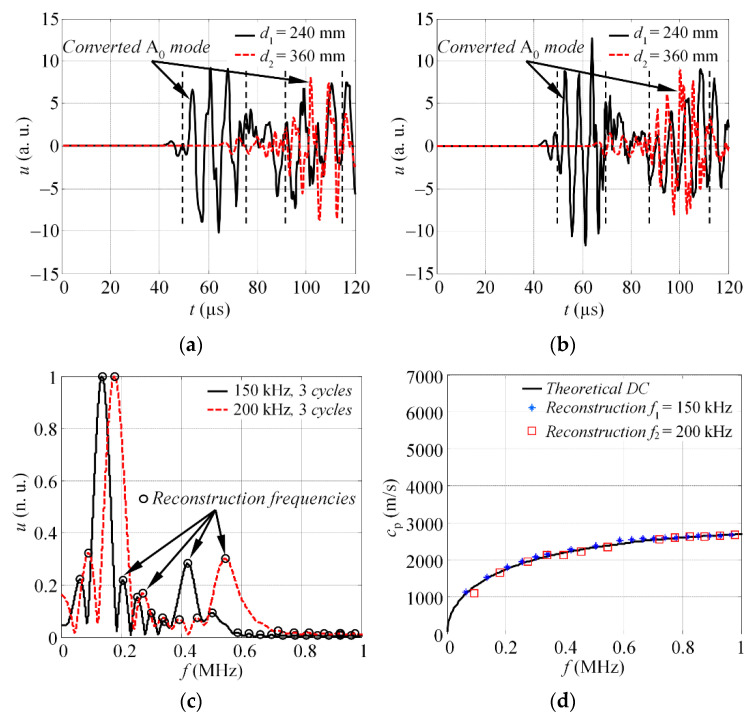
The simulated waveforms of the converted A_0_ mode, at distances *d*_1_ and *d*_2_, in case of (**a**) 150 and (**b**) 200 kHz excitation; (**c**) the magnitude spectra of windowed A_0_ mode at different excitation frequencies; (**d**) the combined reconstruction of phase velocity dispersion curve along with the theoretical estimation.

The simulated waveforms of the converted A_0_ modes, at distances *d*_1_ and *d*_2_, in case of *f*_1_ = 150 kHz and *f*_2_ = 200 kHz excitation, are presented in Figure 9a,b. The selected time windows to cut the wave packet of single mode are indicated with vertical dashed lines. The magnitude spectra of windowed A_0_ mode, at frequencies *f*_1_ and *f*_2_, along with indicated reconstruction frequencies, can be seen on Figure 9c. Finally, the comparison of estimated DC, with the theoretical calculations, is shown on Figure 9d. The results demonstrate a good match between the estimated results and theoretical phase velocities, calculated with the SAFE method. The standard deviation of the reconstructed velocities is equal to *σ* = 47.3 m/s. Overall, the *K* = 32 velocity values were extracted, while 20 (63%) of them were within the range of standard deviation. Even though the number of reconstruction points is less than from the experiments present in previous section, Figure 9d suggests that its quite sufficient for the reconstruction of the segment of dispersion curve. The proposed approach is not limited with two excitation frequencies; hence, the number of reconstruction points can be increased if the segment of dispersion curve is not represented properly.

As it was mentioned previously, the time window selection ambiguity is quite essential in the success of phase velocity reconstruction using phase-shift method, especially for overlapped modes. Hence, the selected time window must hold the single mode only. For complex structures, where signals undergo many reflections, the reconstruction can be quite uncertain. On the other hand, it will be demonstrated in the next chapter that phase velocities can be estimated using part of the signal only. In such a case, the position of the time window must be optimised, i.e., by solving a minimisation problem, to get reasonable velocity reconstruction results.

## 5. Analysis of Multimodal Signals in Anisotropic Structures

**Figure 10:**
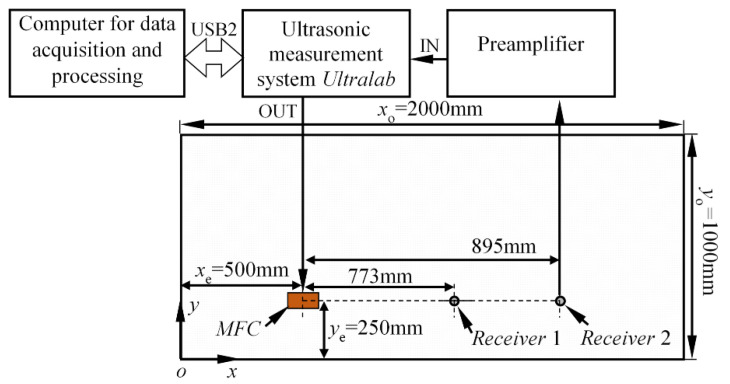
The schematic diagram of the experiments for validation of phase velocity reconstruction approach.

In this section, the performance of the proposed phase velocity reconstruction approach is validated qualitatively by analysing the experimental multimodal signals in an anisotropic structure. For this purpose, the experiments have been carried out in a pitch-catch configuration on the 6-ply GFRP plate (biaxial: 0° and 90°/bias: ±45°/biaxial: 0° and 90°), with dimensions *x*_o_ = 2000, *y*_o_ = 1000, and 4 mm thickness (see Figure 10).

**Figure 11:**
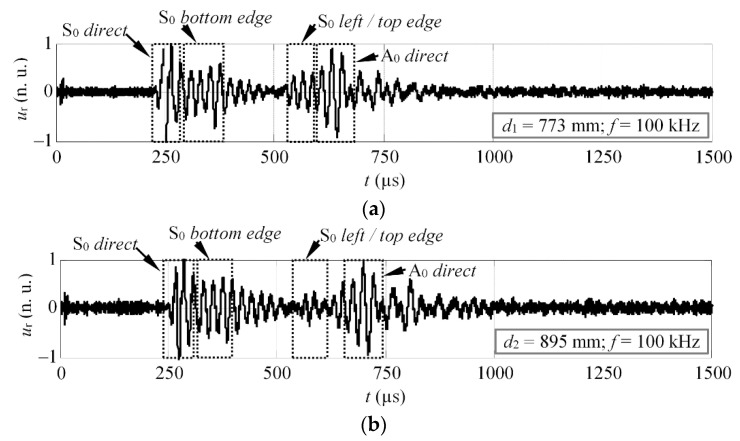
The experimental waveforms obtained on the GFRP sample along the propagation path, at distances (**a**) *d*_1_ and (**b**) *d*_2_.

The Lamb waves were generated using the MFC transducer, centred at the coordinates *x*_e_ = 500 mm, *y*_e_ = 250 mm. It was bonded to the surface of the specimen using a thin layer of gasket maker. The emitter was excited by a three-cycle square pulse, with a central frequency of 100 kHz, where the fundamental A_0_ and S_0_ modes exist in the structure. In this case, the measurements were recorded at a single excitation frequency. Two waveforms were recorded along the wave path (0° propagation), at the distances *d*_1_ = 773 mm and *d*_2_ = 895 mm from the source of Lamb waves (see Figure 10). The proposed phase velocity estimation method was used to extract velocities of the four wave packets: direct S_0_, bottom reflected S_0_, left top edge reflected S_0_, and direct A_0_ mode. The experimentally obtained waveforms, at the distances *d*_1_ and *d*_2_, are presented in Figure 11a,b. The start and stop points of the time windows used to crop the wave packets are indicated by dashed squares.

**Figure 12:**
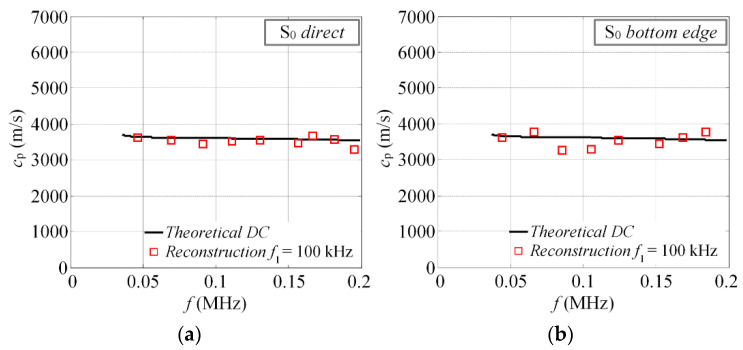

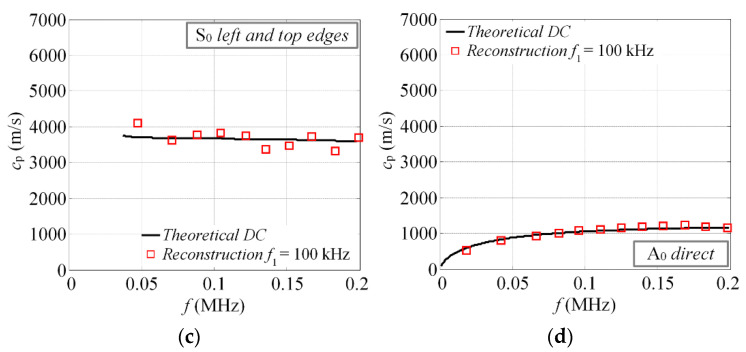
The reconstructed phase velocities of the S_0_ and A_0_ modes: (**a**) S_0_ direct; (**b**) S_0_ bottom edge reflected; (**c**) S_0_ left and top edge reflected; (**d**) A_0_ direct.

The reconstructed phase velocities of different reflections can be seen in Figure 12a–d. The standard deviations for each case of reconstruction are summarized in Table 1. Note that the reconstructed velocity values below 40 kHz were not considered in the calculations of STD.

The results presented above (Figure 12) were found to be in quite good agreement with the theoretical calculations. It suggests that the proposed technique can be used with a certain reliability to extract the phase velocities of GW and identify modes in complex signals. The results show that the velocities of the direct modes are closer to the theoretical values, in comparison to the reflected ones. The average deviation for the direct modes (A_0_ and S_0_) is approximately 75 m/s, while for the reflected S_0_ modes, it is 213 m/s. Several factors may influence the reliability of the results, though. First of all, the selected time windows in Figure 11a,b (dashed squares) may give an idea that this procedure is not very straightforward, especially for the reflected modes. As it turns out, in some cases, part of the wave packet has to be cropped to get better velocity estimation. Another important factor is the propagation distance, which varies for modes arriving at different directions. It means that the distance (*d*) has to be predefined for each wave packet separately. If the propagation distance is not known in advance, an additional error will be obtained. The study revealed that the proposed velocity estimation technique gives an approximate relative experimental error of ±4%, in comparison to theoretical predictions. Meanwhile, for the incident modes, the relative error is always less than a ±2.5%. For example, the 2D FFT method gives an error of approximately of 1% [38]. However, in the study above, the authors used a set of 64 time series, spatially sampled at 1 mm, to achieve such accuracy.

## 6. Conclusions

In this paper, a phase velocity reconstruction approach, based on the phased spectrum method, was developed, which exploits several excitation frequencies of the ultrasonic probe and estimates phase velocity values at peak frequencies of the magnitude spectra. The proposed approach allows us to reconstruct phase velocities with high accuracy in wide frequency bandwidth using only two waveforms measured at close proximity. In contrast to the classic phase spectrum method, the proposed technique offers an increased reconstruction bandwidth (from −6 to −20 dB) and reduced relative error of phase velocity reconstruction (from ±11% to ±4%). The main outcomes of the research can be summarized as follows:

- It was found that the accuracy of the classic phase spectrum method can be improved if several frequencies are used to drive the transducer, while the phase velocities are reconstructed at peak values of Fourier spectra only. Such an approach allows us to avoid low energy frequency components, where the velocity estimation error is likely to increase.
- The initial experiments demonstrated that the proposed phased spectrum method can increase the reconstruction bandwidth, from −6 to −20 dB, of the sensor and improve the standard deviation of velocity reconstruction, from 592 to 161 m/s. For the experimental S_0_ mode, this results in a velocity estimation relative error improvement, from ±11% to ±3%.
- The finite element simulations demonstrated the applicability of the proposed approach in detecting converted guided wave modes. It was demonstrated that the phase velocities of converted modes can be reconstructed with a standard deviation of 47.3 m/s, even if the modes are partly overlapped with direct waves.
- Finally, the proposed method was demonstrated to be appropriate for the analysis of complex guided wave signals, with multiple co-existing modes. It was estimated that average deviation for the direct modes (A_0_ and S_0_) is approximately 75 m/s, while for the reflected S_0_ modes, it is 213 m/s. While analysing the overlapped complex guided wave signals, the proper selection of time gate is the most important parameter for the accuracy of reconstruction.
- It was estimated that the average phase velocity reconstruction error of the proposed method, including both symmetrical and asymmetrical modes, is up to ±4%. The classic phase spectrum method provides an approximate reconstruction error of ±11%. Other techniques, reported in the literature, can achieve velocity reconstruction with an average error of ±1%; however, at least 64 signals need to be acquired to achieve such accuracy.

## Figures and Tables

**Table 1:** The standard deviation of the estimated phase velocities for different GW mode packets.

Type of Mode	Velocity Standard Deviation, *σ* (m/s)
S_0_ direct	97.4
S_0_ bottom edge	202.7
S_0_ left and top edge	224.5
A_0_ direct	51.5
